# Longitudinal [^18^]UCB-H/[^18^F]FDG imaging depicts complex patterns of structural and functional neuroplasticity following bilateral vestibular loss in the rat

**DOI:** 10.1038/s41598-022-09936-w

**Published:** 2022-04-11

**Authors:** Melissa Antons, Magdalena Lindner, Maximilian Grosch, Rosel Oos, Giovanna Palumbo, Matthias Brendel, Sibylle Ziegler, Peter Bartenstein, Marianne Dieterich, Andreas Zwergal

**Affiliations:** 1 German Center for Vertigo and Balance Disorders, DSGZ, University Hospital of LMU Munich, 81377 Munich, Germany; 2 Department of Nuclear Medicine, University Hospital of LMU Munich, 81377 Munich, Germany; 3 Department of Neurology, University Hospital of LMU Munich, 81377 Munich, Germany; 4 Munich Cluster of Systems Neurology, SyNergy, Munich, Germany

**Keywords:** Neural circuits, Regeneration and repair in the nervous system, Sensorimotor processing, Synaptic plasticity

## Abstract

Neuronal lesions trigger mechanisms of structural and functional neuroplasticity, which can support recovery. However, the temporal and spatial appearance of structure–function changes and their interrelation remain unclear. The current study aimed to directly compare serial whole-brain in vivo measurements of functional plasticity (by [^18^F]FDG-PET) and structural synaptic plasticity (by [^18^F]UCB-H-PET) before and after bilateral labyrinthectomy in rats and investigate the effect of locomotor training. Complex structure–function changes were found after bilateral labyrinthectomy: in brainstem-cerebellar circuits, regional cerebral glucose metabolism (rCGM) decreased early, followed by reduced synaptic density. In the thalamus, increased [^18^F]UCB-H binding preceded a higher rCGM uptake. In frontal-basal ganglia loops, an increase in synaptic density was paralleled by a decrease in rCGM. In the group with locomotor training, thalamic rCGM and [^18^F]UCB-H binding increased following bilateral labyrinthectomy compared to the no training group. Rats with training had considerably fewer body rotations. In conclusion, combined [^18^F]FDG/[^18^F]UCB-H dual tracer imaging reveals that adaptive neuroplasticity after bilateral vestibular loss is not a uniform process but is composed of complex spatial and temporal patterns of structure–function coupling in networks for vestibular, multisensory, and motor control, which can be modulated by early physical training.

## Introduction

Neuroplasticity following neuronal lesions is an inherent resource of the brain to augment functional recovery^1,2^. Multiple mechanisms evolving on different temporal scales are engaged, starting with rapid onset alterations during a critical post-lesion time period and followed by slower processes of reorganization, which may sustain functional gains or losses^3,4^. At the neuronal level, changes in membrane excitability, synaptic, dendritic and axonal anatomy have been described both in animals and humans^5,6^. At the network level, recruitment of novel or alternative neural circuits and changes in the strength of connections between brain areas may foster adaptation to functional consequences of neuronal damage^7,8^. Previous studies have targeted the cerebral cortex to investigate adaptive plasticity. However, it is widely acknowledged that neural substrates of functional recovery are distributed over multiple sites at different brain levels and reorganization requires fine-tuned activity at cortical and subcortical sites^1,9^.

Temporal dynamics and spatial coherence of structure–function relationships after a neuronal injury are not fully understood yet. Theoretically, the lesion-induced change in function could either be succeeded by a structural alteration in corresponding brain regions (‘form follows function’)^10^, or an adaptive reorganization of network structures could induce a possible gain of function (‘function follows form’). The latter would resemble a developmental strategy, where structural plasticity generates new functional specialization^11^.

Sensory deprivation models have been used extensively to investigate plasticity mechanisms, for example, in cortical representations^12–14^. In this line, animal models of inner ear damage are well suited to investigate lesion-induced cerebral plasticity in correlation to behavioural markers^15^. Bilateral vestibular deafferentation in rodents results in a typical clinical syndrome with gait ataxia and postural imbalance, which partially recovers over weeks due to adaptation and sensory substitution^16–18^. In contrast, spatial orientation deficits persist over time^19^. Structural changes such as altered receptor expressions, synaptic morphology and cell proliferation were reported in vitro in the brainstem, striatum, hippocampus, and frontal cortex after bilateral vestibular deafferentation in the rat^17,20–22^. In clinical practice, multimodal physical training of eye-head-coordination, postural balance, and locomotion are established and evidence-based treatment principles for patients with bilateral vestibular loss^23^. While these exercises are effective at improving mobility and reducing the risk of falls, their mode of action on cerebral structure and function is barely understood.

In the current dual tracer study, we performed serial [^18^F]UCB-H and [^18^F]fluorodeoxyglucose positron emission tomography ([^18^F]FDG-PET) measurements from baseline to 9 weeks after bilateral labyrinthectomy (BL) in the rat, to directly compare post-lesion whole-brain changes in synaptic density and glucose metabolism and investigate the effect of immediate locomotor training on adaptive neuroplasticity. In this model, [^18^F]FDG uptake was interpreted as an equivalent of functional plasticity mechanisms (e.g., change in neuronal activity), while [^18^F]UCB-H binding was taken as a biomarker for structural remodelling (e.g., synaptic loss or synaptic sprouting)^24^. We hypothesized that (1) dynamic changes of structure and function would appear at multiple brain levels with a focus on ascending vestibular networks^25,26^, as well as cortical and subcortical sensorimotor networks involved in sensory substitution^27^ and locomotor adaptation^28^; (2) complex patterns of structure–function coupling would be found across different neural networks, which potentially represent distinct modes of lesion-induced plasticity (i.e., loss/gain of function); (3) locomotor training might augment the process of adaptive plasticity and partially improve behavioural deficits after BL. To our best knowledge, this study is the first to apply [^18^F]UCB-H longitudinally and in comparison to [^18^F]FDG to investigate whole-brain adaptive plasticity in vivo following a neuronal lesion.

## Results

### Statistical analysis of mean normalized activity in segmented brain regions in all animals

**Figure 1:**
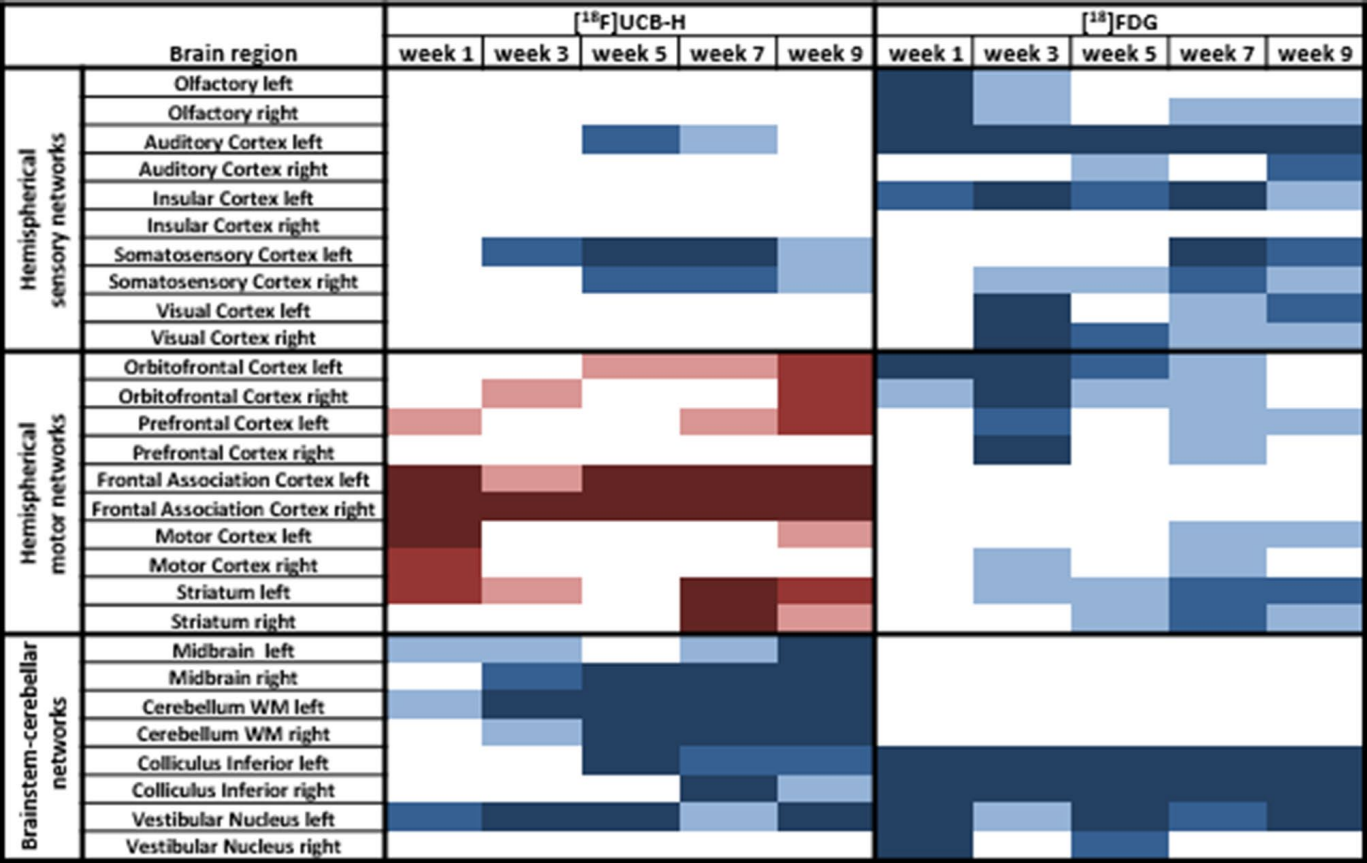
Overview of the changes in synaptic density measured by [^18^F]UCB-H PET (left side) and regional cerebral glucose metabolism (rCGM) measured by [^18^F]FDG PET (right side) in 28 selected brain regions, which are known to be involved in vestibular and sensorimotor processing. Mean normalized activity values per segmented brain region at 1, 3, 5, 7 and 9 weeks post bilateral labyrinthectomy (BL) were statistically compared using a one-way repeated measurement ANOVA with post-hoc paired t-tests and Bonferroni correction for multiple testing. Significant increases in synaptic density or rCGM are visualized in red scales (light red: *p* < 0.05, red: *p* < 0.005, dark red: *p* < 0.001), decreases in blue scales (light blue: *p* < 0.05, blue: *p* < 0.005, dark blue: *p* < 0.001). Comparison of [^18^F]UCB-H and [^18^F]FDG patterns shows a complex structure–function relationship across brain networks. [^18^F]FDG: [^18^F]fluorodeoxyglucose.

Mean normalized activity values per segmented brain region at 1, 3, 5, 7, 9 weeks post BL were compared to baseline in all rats separately for [^18^F]UCB-H and [^18^F]FDG to delineate spatial and temporal patterns of changes in synaptic density and regional cerebral glucose metabolism (rCGM). In general, synaptic density decreased significantly in 16.7% and increased in 15% of all brain regions post BL. In contrast, rCGM decreased in 33.3% of all regions, but increased in none. In terms of anatomical distribution, serial [^18^F]UCB-H measurements indicated a main cluster with an overall decrease of synaptic density in brainstem-cerebellar networks (e.g., vestibular nuclei, cerebellar white matter, colliculus inferior, midbrain) (Fig. 1). Brain regions with a synaptic increase clustered in the orbitofrontal, prefrontal, and frontal association cortex (mean onset 1–5 weeks post BL), as well as in the striatum (7 and 9 weeks post BL). Sensory cortical areas showed a diverse pattern with a decreased binding in the auditory and somatosensory cortex after week 3 post BL, whereas visual cortex showed no change at any time point. Spatial patterns of [^18^F]FDG uptake showed clusters of early-onset rCGM decrease in the brainstem (vestibular nuclei, colliculus inferior), orbitofrontal, olfactory, auditory and insular cortex from 1 week post BL, and later rCGM decrease in other sensory cortical regions (including the visual, somatosensory cortex from 3 weeks post BL), as well as in the motor cortex and striatum (from 3 weeks post BL).

### Statistical voxel-wise whole-brain analysis in all animals

**Figure 2:**
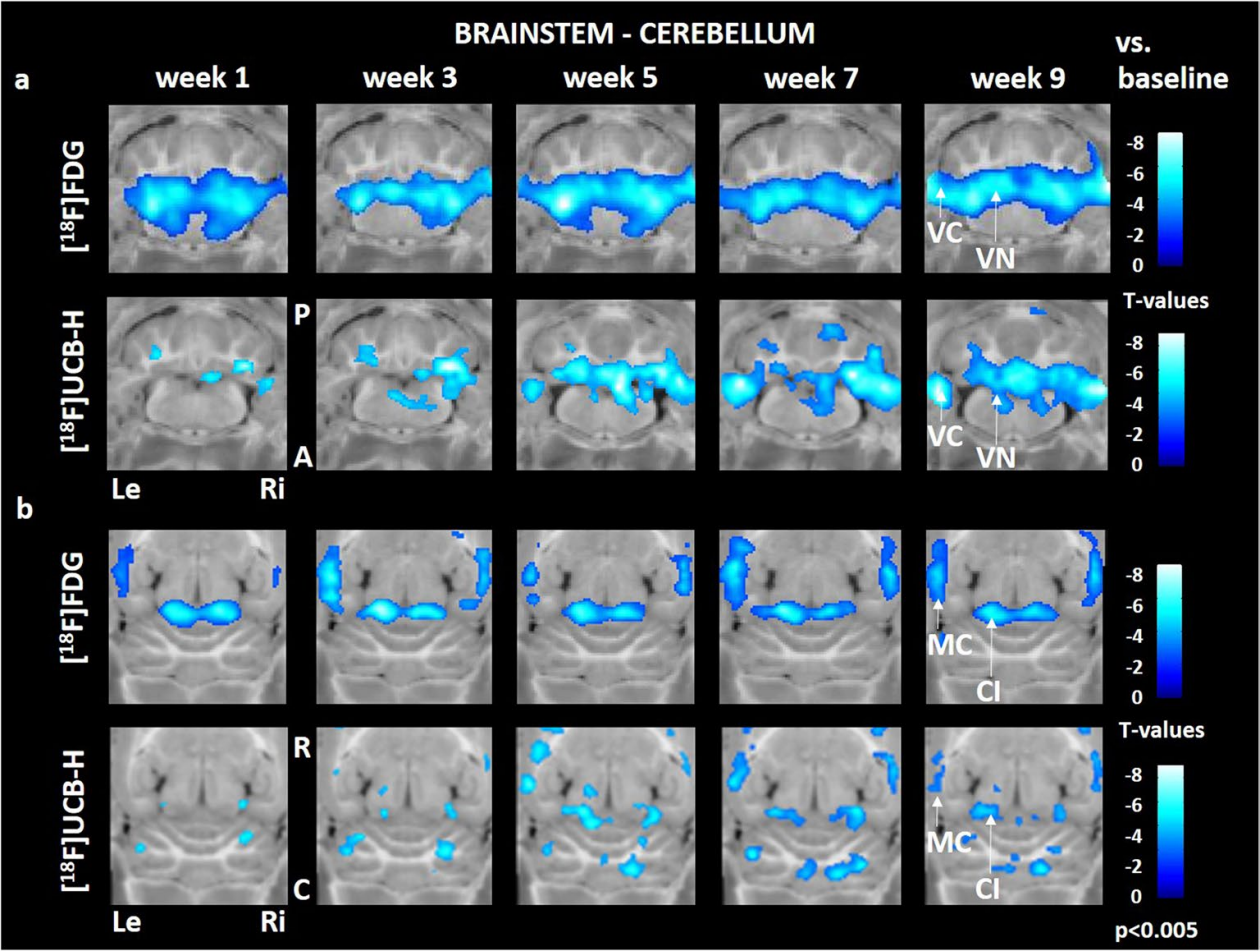
Comparison of regional cerebral glucose metabolism and synaptic density in the brainstem, cerebellum and multisensory cortex. Early decrease in [^18^F]FDG uptake is followed by reduced [^18^F]UCB-H binding in the vestibular nuclei, adjacent vestibular cerebellum (**a**), colliculus inferior and multisensory cortex (**b**). C: caudal, R: rostral, Le: left, Ri: right, CI: colliculus inferior, MC: multisensory cortex, VC: vestibular cerebellum, VN: vestibular nucleus, [^18^F]FDG: [^18^F]fluorodeoxyglucose. Changes against baseline are depicted as t-values at a significance level of *p* < 0.005.

A marked decrease of rCGM (compared to baseline) started in the vestibular nuclei and adjacent vestibular cerebellum in week 1 and persisted up to week 9 post BL. In comparison, loss of synaptic density was observed with a temporal delay, beginning at 3 weeks and progressing until 9 weeks post BL (Fig. 2a). Similar dynamics were found in the colliculus inferior, which display a reduced [^18^F]FDG uptake from week 1–9 post BL, while [^18^F]UCB-H binding decreased progressively from week 3 post BL. Parietal multisensory cortex areas showed an early rCGM decrease (week 1) and a delayed loss in synaptic density (week 3) (Fig. 2b).

**Figure 3:**
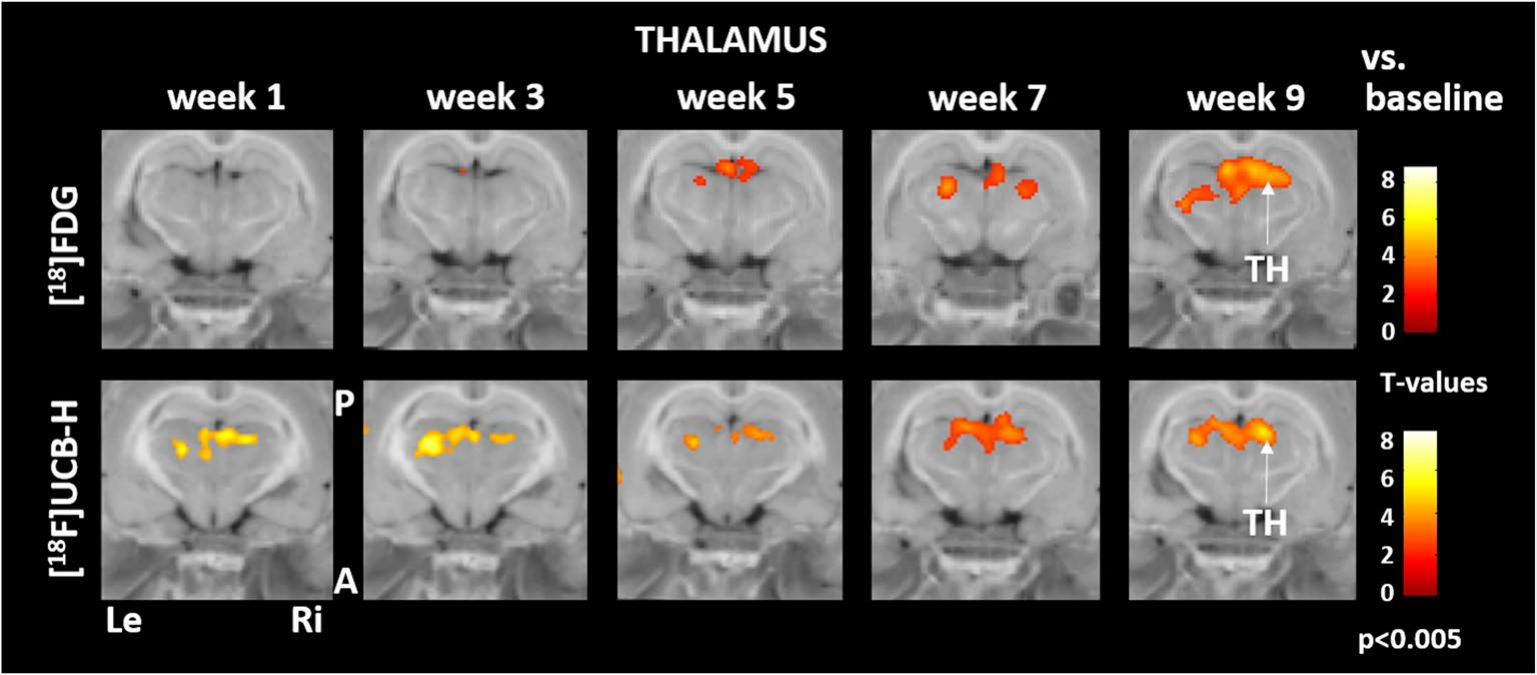
Comparison of regional cerebral glucose metabolism and synaptic density in the thalamus. Early increase of synaptic density in the posterolateral thalamus was succeeded by more [^18^F]FDG uptake during weeks 5 to 9 post BL. A: anterior, P: posterior, Le: left, Ri: right, TH: thalamus, [^18^F]FDG: [^18^F]fluorodeoxyglucose. Changes against baseline are depicted as t-values at a significance level of *p* < 0.005.

Synaptic density in the posterolateral thalamus increased progressively from week 1 to 9 post BL. [^18^F]FDG uptake started to increase with a temporal delay at week 5 post BL at the same anatomical location and further advanced until week 9 post BL (Fig. 3).

**Figure 4:**
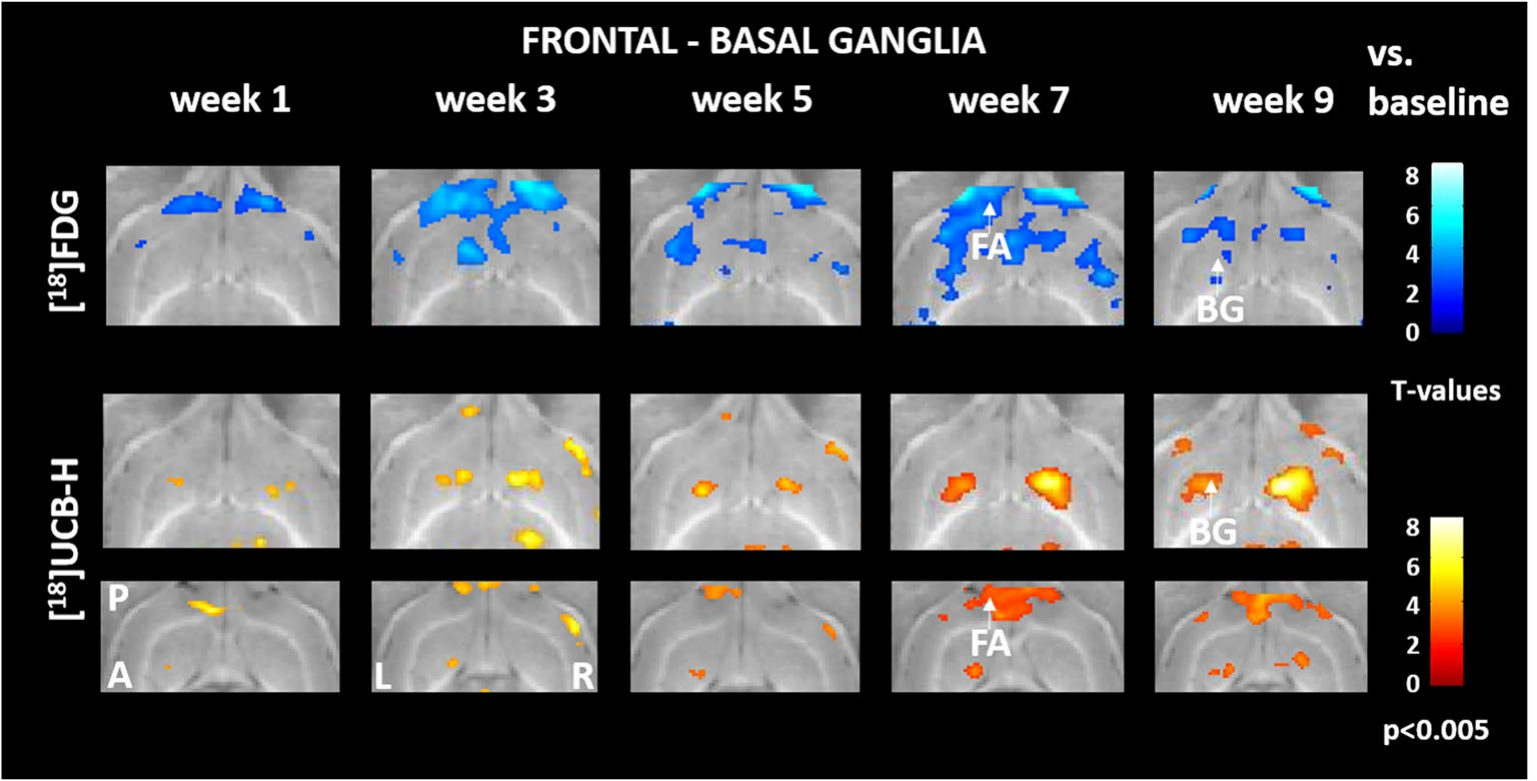
Comparison of regional cerebral glucose metabolism and synaptic density in frontal–basal ganglia networks. Reduced [^18^F]FDG uptake in the frontal association cortex and striatum was paralleled by an increase in [^18^F]UCB-H binding. A: anterior, P: posterior, Le: left, Ri: right, BG: basal ganglia, FA: frontal association cortex, [^18^F]FDG: [^18^F]fluorodeoxyglucose. Changes against baseline are depicted as t-values at a significance level of *p* < 0.005.

In the frontal cortex-basal ganglia loops, voxel-based analysis showed an inverted pattern of [^18^F]FDG uptake and [^18^F]UCB-H binding. While rCGM in the frontal association cortex was reduced from week 1 post BL, synaptic density increased after week 3 post BL. In parallel, striatal rCGM decreased after week 3 post BL, while synaptic density bilaterally increased in this brain region (Fig. 4).

### Statistical voxel-wise analysis of training and no training group

**Figure 5:**
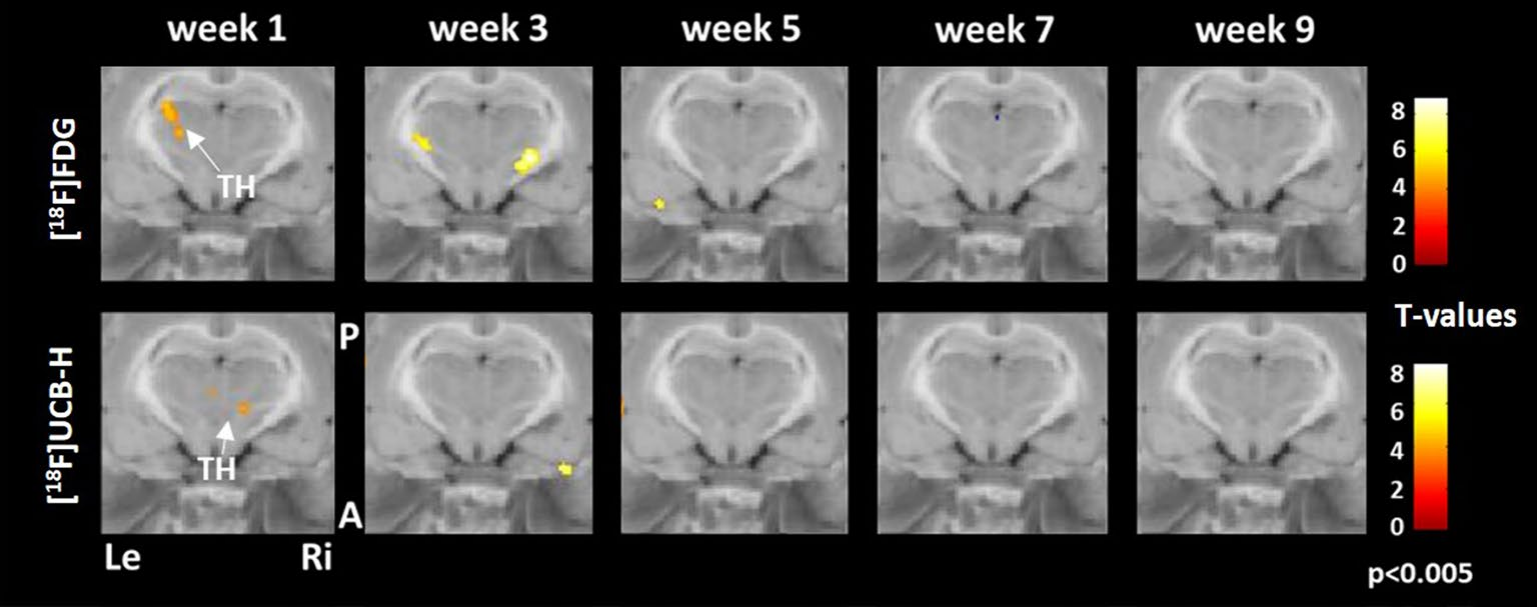
Comparison of regional cerebral glucose metabolism and synaptic density in the thalamus in the training and no training groups. Increased [^18^F]FDG uptake in the thalamus was paralleled by an increase in [^18^F]UCB-H binding in the training group. A: anterior, P: posterior, Le: left, Ri: right, TH: thalamus, [^18^F]FDG: [^18^F]fluorodeoxyglucose. Changes between groups are depicted as t-values at a significance level of *p* < 0.005.

In the thalamus, voxel-based analysis showed a significantly higher uptake of [^18^F]FDG and [^18^F]UCB-H in the training group. Synaptic density increased in week 1 and rCGM in week 1 and week 3 post BL (Fig. 5).

### Behavioural testing

All rats showed characteristic signs of bilateral vestibular differentiation, such as postural imbalance, gait ataxia, head instability (‘bobbing’) and opisthotonus. On clinical assessment, postural imbalance and gait ataxia became less apparent after day 5 post BL and head bobbing stopped completely until day 5 post BL. Phases with opisthotonus persisted over the course of the experiment but were observed less frequently with time. Analysis of locomotion in the open field indicated that velocity increased significantly and progressively from day 3 post BL compared to baseline (n = 22, ANOVA, F = 19.59, post-hoc comparison: day 3: *p* = 0.006, day 5: *p* = 6.40 E−05, week 1: *p* = 7.57 E−07, week 3: *p* = 3.01 E−06, week 5: *p* = 2.31 E−05, week 7: *p* = 2.76 E−05, week 9: *p* = 1.57 E−05, see Supplementary Fig. S1).

**Figure 6:**
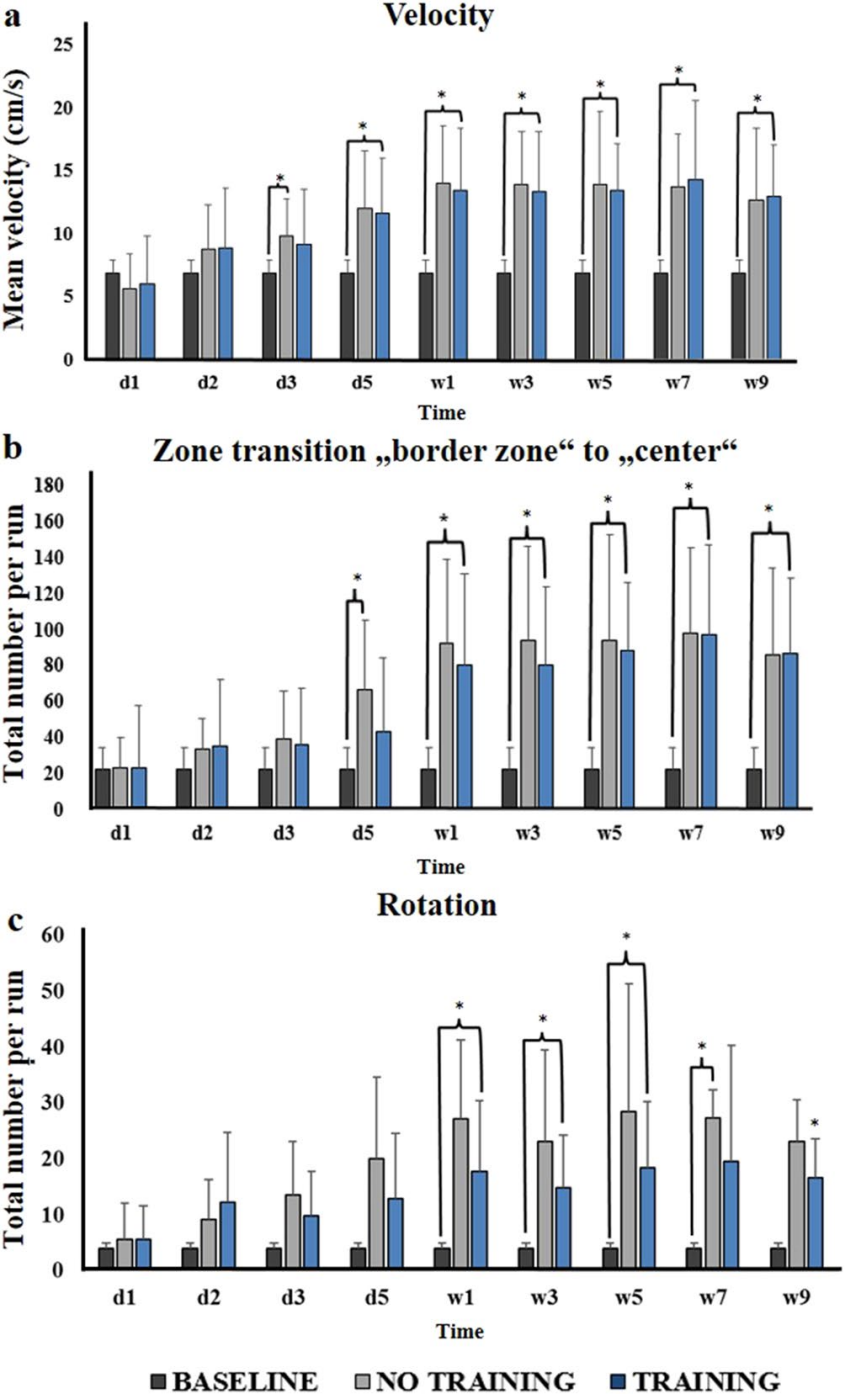
Behavioural parameters in the open field. (**a**) Locomotor velocity for no training and training subgroups from baseline to 9 weeks post BL. (**b**) Total number of zone transition from border to center zone for the no training and training subgroups at different time points post BL compared to baseline. (**c**) Total number of rotations per run for no training and training subgroups at different time points post BL. Values are depicted as mean + standard deviation. Significant differences (*) are derived from an ANOVA with post-hoc testing and Bonferroni correction at a level of *p* < 0.05. d: day, w: week.

Analysis of subgroups (training and no training, n = 11 each) showed a significant increase in locomotor velocity from day 5 post BL compared to baseline in both subgroups (ANOVA, training group: F = 10.77, post-hoc comparison: day 5: *p* = 0.01, week 1: *p* = 0.001, week 3: *p* = 0.005, week 5: *p* = 0.0006, week 7: *p* = 0.02, week 9: *p* = 0.01; no training group: F = 8.22, post-hoc comparison: day 5: *p* = 0.02, week 1: *p* = 0.003, week 3: *p* = 0.003, week 5: *p* = 0.04, week 7: *p* = 0.01, week 9: *p* = 0.04). No relevant differences relative to each other were found for locomotor velocity at any time point post BL (Fig. 6a). The number of zone transitions between the border and center zone of the open field increased significantly from week 1 post BL compared to baseline in both subgroups (ANOVA, training group: F = 15.18, post-hoc comparison: week 1: *p* = 0.009, week 3: *p* = 0.004, week 5: *p* = 0.0002, week 7: *p* = 0.003, week 9: *p* = 0.004; no training group: F = 13.66, post-hoc comparison: week 1: *p* = 0.002, week 3: *p* = 0.002, week 5: *p* = 0.008, week 7: *p* = 0.001, week 9: *p* = 0.01) (Fig. 6b). The number of rotations around the body axis per run increased significantly in both subgroup from week 1–5 post BL (ANOVA, training group: F = 4.87, post-hoc-comparison: week 1: *p* = 0.02, week 3: *p* = 0.008, week 5: *p* = 0.01; no training group: F = 6.61, post-hoc comparison: week 1: *p* = 0.002, week 3: *p* = 0.03, week 5: *p* = 0.05) (Fig. 6c). After day 5 post BL rats in the no training subgroup displayed more rotations than in the training subgroup.

## Discussion

The major findings of this study were as follows: (1) the combination of [^18^F]UCB-H and [^18^F]FDG measurements was a feasible approach to depict dynamic changes of structure and function following bilateral vestibular loss in the rat; (2) lesion-induced plasticity was not a uniform process, but involved complex structure–function relationships at various brain levels with different temporal scales; (3) a ‘form follows function’ type of plasticity was found in brainstem and cerebellar networks, which were most closely exposed to loss of vestibular input, while a ‘function follows form’ type of plasticity appeared in thalamic nuclei dedicated to multisensory processing and motor adaptation; (4) locomotor training had a mild effect on dynamic balance after BL, which was paralleled by changes in synaptic density and glucose metabolism in the thalamus.

### [^18^F]UCB-H/[^18^F]FDG dual tracer PET imaging: a novel method to study neuroplasticity

Advances in non-invasive measurements, such as magnetic resonance imaging (MRI), PET, or electroencephalography (EEG), have massively contributed to our understanding of adaptive brain plasticity in vivo following neuronal lesions. While MRI has an excellent spatial resolution, EEG has a better temporal resolution. MRI techniques may provide information on the relation of structural changes in grey and white matter (e.g., voxel-based morphometry, diffusion-tensor imaging)^29–33^, and functional activity of brain networks (e.g., resting-state functional MRI) during behavioural recovery processes after a neuronal lesion^34^. However, the cellular mechanisms underpinning adaptive structural changes in grey and white matter are not completely understood yet^1^.

Molecular imaging may be complementary in this respect, especially since novel tracers targeting synaptic vesicular protein 2A (SV2A such as [^11^C]UCB-J or [^18^F]UCB-H) are available to quantify synaptic density as a surrogate for axonal structure^24,35,36^, which can be compared to ‘classical’ markers of neuronal activation, such as [^18^F]FDG. Recently, it was shown that the [^11^C]UCB-J signal was not affected by short-term changes in neuronal activity during an activation task^37,38^. On the other hand, short-term dynamics in [^18^F]UCB-H binding were depicted in a temporal lobe epilepsy model, which depended on the disease stage^39^. In a mouse model of Alzheimer disease, therapeutic effects of a disease-modifying drug could be visualized and quantified by [^18^F]UCB-H-PET imaging^40^. Clinical studies in patients with epilepsy or dementia syndromes have shown that SV2A tracers can reliably detect a loss of synaptic density, which is associated with neuronal damage^41,42^. In a rat model of Parkinson’s disease, complex cortical changes of synaptic density and neuronal metabolic function have recently been described by [^11^C]UCB-J/[^18^F]FDG PET imaging^43^. However, to the best of our knowledge a dual tracer approach using serial [^18^F]UCB-H/[^18^F]FDG measurements has not been applied to study time dynamics and spatial distribution of neuroplasticity induced by acute vestibular lesions. In this study, we strived to depict changes in synaptic density and regional glucose consumption longitudinally in an established sensory deprivation model in the rat. For this methodological purpose, subgroups with and without training were pooled to increase statistical robustness. We think that this approach is reasonable, because subgroups were comparable for most behavioural parameters (see below) and underwent an identical longitudinal imaging protocol. We were able to show dynamic changes of synaptic density across six consecutive imaging time points within 10 weeks, which appeared in biologically plausible networks in the brainstem, cerebellum, thalamus, multisensory cortex and motor-basal ganglia circuits. Most importantly, rCGM changes were found in similar regions, but with different temporal scales. The direct comparison of time courses and spatial distribution of [^18^F]UCB-H and [^18^F]FDG signals may potentially allow conclusions to be drawn about the mode of plasticity (‘form follows function’ or ‘function follows form’).

### Differential modes of structural and functional plasticity following complete vestibular loss

The current study shows three major modes of structure–function coupling following bilateral vestibular loss, which appeared in different neural networks:

1. In brainstem-cerebellar networks, early-onset decrease in rCGM after BL was followed by reduced synaptic density by mean 3 weeks later in the same brain regions (Fig. 3). A rapid drop in neuronal activity in the vestibular and cochlear nucleus is expected after BL, because these nuclei receive primary afferents from the inner ear and are consequently affected most by loss of signal input^44^. Of note, rCGM also decreased in secondary hubs of vestibular and auditory processing (e.g., vestibular cerebellum, colliculus inferior) instantaneously after BL. We think that functional changes of central auditory networks (e.g., cochlear nucleus, colliculus inferior) reflect some concomitant damage of toxic BL to either the cochlear hair cells or alternatively an impairment of sound conduction by damage to the tympanic membrane. Of note, in a mouse model of arsanilate-induced labyrinthectomy no significant damage was found to the hair cells of the cochlea and stria vascularis, but an elevated threshold of auditory brainstem responses due to paracentesis of the tympanic membrane was detected^45^. Damage to inner ear inputs resulted in a delayed loss of synaptic density in the same regions (vestibular nuclei, vestibular cerebellum, colliculus inferior), which slowly increased until 9 weeks post BL. This observation can be interpreted as a persistent structural degeneration in brainstem-cerebellar networks following irreversible loss of bilateral vestibular function. Our data seem to be in partial contrast to a previous in vitro study that showed a loss of synaptic density of about 35% in the medial vestibular nucleus 1 week after unilateral vestibular neurectomy in the cat, which was followed by a synaptic reoccupation from 3 weeks to 5 months after the lesion^46^. However, it has to be considered that static behavioural deficits after a unilateral vestibular lesion recover rapidly and completely due to central vestibular compensation, while a bilateral vestibular lesion induces symptoms that are more persistent and can in part not be fully compensated. It seems likely that the intact contralateral vestibular inputs contribute to synaptic reoccupation in unilateral vestibular lesions. Furthermore, a complete vestibular differentiation by neurectomy of the vestibular nerve may cause more severe changes of synaptic density compared to a pure loss of vestibular hair cells induced by chemical labyrinthectomy.
2. In the thalamus, synaptic density started to increase bilaterally from 1 week after BL, which was succeeded by an rCGM increase 2 weeks later (Fig. 4). The thalamus is a well-known hub for integration of multisensory inputs from the vestibular, the visual, and the somatosensory system, as well as vestibular-motor interaction^47^. Various thalamic subnuclei with a functional role in multisensory processing (ventroposterior nuclei)^48^, motor control (ventrolateral nuclei)^49^, heading direction and spatial orientation (anterior nuclei)^50^ receive ascending vestibular information directly from the vestibular nuclei or from vestibular cerebellar nuclei^25,26,51,52^. Changes in synaptic density and glucose metabolism in the posterior and lateral parts of the thalamus found in the current study likely represent a process of adaptive plasticity, which contributes to multisensory substitution and recalibration. Accordingly, human neuroimaging studies have documented changes in cortical and subcortical multisensory networks in patients with chronic bilateral vestibular loss, which suggest an increased visual substitution^53,54^. From a neuroscientific perspective, it is interesting that synaptic density in the thalamus increases before glucose consumption. It seems that during adaptive neuroplasticity novel synaptic connections are established first, which in turn result in a gain of function and increase of neuronal activity. This example shows that function can follow form, similar to the processes in the developing brain during childhood^1^. This poses the question, whether the brain reactivates these developmental strategies in the case of a neuronal lesion. Interestingly, dynamic balance, which can be estimated by the number of body rotations, deteriorated until week 3 post BL and stabilized thereafter. This may be interpreted as a behavioural correlate of adaptive plasticity driven by the thalamus.
3. In frontal-basal ganglia loops, we found an increase of synaptic density, which was paralleled by a relative rCGM decrease especially after 3 weeks post BL (Fig. 5). Frontal-basal ganglia networks are critically involved in supraspinal locomotor control^28,55^. An established concept of basal ganglia control of movement states that a direct pathway via striatal medium spiny neurons (dMSNs) expressing dopamine D1 receptor facilitates movements, while an indirect pathway marked by iMSNs expressing dopamine D2 and adenosine 2a receptors suppresses movement^56–58^. The same model seems to apply for locomotor control. Stimulation of dMSNs increases and of iMSNs suppresses locomotion by downstream control of the mesencephalic locomotor region (MLR)^55,59^. Indeed, connections between the vestibular system and the basal ganglia have been reported^49^. Neuronal projections from the medial vestibular nucleus via the parafascicular nucleus of the thalamus to the dorsolateral putamen were documented by tracing experiments in the rat^60^. In another study, neurochemical changes in the striatum were induced by vestibular stimulation^61^. Furthermore, vestibular signals may project directly to the MLR and thereby modulate the locomotor pattern^62,63^. It is a striking and pathognomonic behavioural feature of bilateral vestibular loss in rats that animals develop a persistent hyperlocomotion pattern^64,65^. Behavioural data in our rat model exactly resembled this known phenomenon (Fig. 6). It has been suggested that loss of vestibular input to the striatum may hamper the balance of dMSN and iMSN in favor of the direct pathway, thus activating locomotion^49^. From a theoretical point of view, increased locomotion following bilateral vestibular loss could be due to an upregulation of dMSNs or a downregulation of iMSNs. Changes of D2 receptor expression in the striatum were not identified following BL in the rat^66^. However, patients with chronic bilateral vestibular loss had a reduced D2/D3 receptor availability in the striatum bilaterally, which also correlated to their handicap^67^. How can the findings of the current [^18^F]UCB-H/[^18^F]FDG tracer study be interpreted in this context? It is striking that frontal-basal ganglia hubs showed a relatively reduced rCGM throughout the experiment. Although [^18^F]FDG data cannot inform about the underlying circuit, in view of the behavioural signature of hyperlocomotion it seems likely that missing vestibular input reduced the locomotor control from the frontal cortex via the indirect basal ganglia pathway activating the direct basal ganglia pathway. The significant increase of synaptic density in the frontal association cortex and striatum could be seen either as an adaptive response to this functional imbalance of direct–indirect pathways, or as a secondary maladaptive effect due to the overactive direct pathway. As the hyperlocomotion does persist after irreversible vestibular loss, it seems that the synaptic plasticity in the basal ganglia is not sufficient to compensate for this behavioural dysregulation.

### Impact of locomotor training on adaptive brain plasticity following bilateral vestibular loss

Multimodal physical training is the mainstay of therapy in patients with bilateral vestibular loss^23,68^. The applied principles are based on neurophysiological considerations and tailored to training of the vestibular-ocular reflex (habituation training), the interplay of multisensory sources to control posture (sensory pertubation training), and dynamic balance (locomotor training)^69,70^. While the effectiveness of these exercises is highly evidence based^71^, their mode of action on cerebral function and structure has been completely neglected so far. Cortical reorganization by a motor training is well documented for patients with stroke^72,73^. From this literature, we have learned that new training methods based on basic neuroscientific principles such as motor imagery^74,75^ or action activation^76^ may add to the therapeutic success. In the current study, we aimed to start with a comparably simple training program based on the voluntary use of running wheels installed in the cages, to understand how this intervention may influence behaviour, brain structure and function after bilateral vestibular loss in the rat. This selection was motivated by the fact that active locomotion stimulates multisensory feedback and trains motor control. Furthermore, a beneficial effect of voluntary running wheel training has been shown in various neurological disease models^77,78^. In the current study, we found an effect of running wheel training on dynamic balance, as measured by the number of body rotations, with a peak 1–5 weeks post BL, while other markers of locomotor behaviour such as velocity or movement duration remained unchanged (Fig. 6). Accordingly, transient and subtle changes of synaptic density and regional glucose metabolism were found in the thalamus (Fig. 5). Given the role of the thalamus for multisensory adaptation and postural control, it is reasonable that the therapeutic effect of running wheel training was mediated by this brain region. Alterations of [^18^F]UCB-H/[^18^F]FDG binding in brainstem-cerebellar circuits and frontal-basal ganglia circuits were not affected by training after BL. The finding of a training effect on adaptive plasticity and balance control in BL is promising, because it gives first evidence for a potential neurobiological correlate of vestibular rehabilitation. However, the rather mild and transient effects documented in this study show that a stereotyped training such as running wheel locomotion likely is not sufficient to induce persistent and functionally relevant effects on balance and locomotor control in a three-dimensional space. Future studies might test the effect of more elaborated training protocols (e.g., visual cueing, vibration feedback, more complex and varying movements) on both adaptive brain plasticity and behavioural recovery in vestibular animal models^79,80^.

In conclusion, the current study shows the potential of a [^18^F]UCB-H/[^18^F]FDG dual tracer application to investigate structural and functional plasticity mechanisms after a neuronal injury and the effect of treatment on this process. Lesion-induced neuroplasticity is a complex process with different modes of structure–function coupling, which arise in various neural networks and at different temporal courses. An improved understanding of these processes could help to tailor interventions to the optimal time window and to target specific networks by therapeutic strategies at different times.

## Methods

### Animals and housing

All animal experiments were approved by the government of Upper Bavaria and performed in accordance with the guidelines for the use of living animals in scientific studies and the German Law for the Protection of Animals (ROB-55.2-2532.Vet_02-21-32). The authors complied with the ARRIVE guidelines.

Male Sprague–Dawley rats (mean weight 400 +/− 20 g, age 10–11 weeks at the time of surgery, Charles River, Sulzfeld) were housed two animals per cage in a temperature- and humidity-controlled room with a 12 h light / dark cycle and free access to food and water. One group of rats (n = 12) was housed in cage systems from IntelliBio® (IntelliBio® Innovations, France) with integrated running wheels. Another group (n = 12) was placed in double decker cages (GR1800, Tecniplast, Germany).

### Running wheels

After baseline measurements, an RFID Chip (IntelliBio®) was transplanted between the shoulder blades of all animals to ensure uniform conditions. The rats were then randomized into two groups (training and no training). The training group could use the integrated running wheels in their cages freely. Use of the running wheel was detected automatically by the implanted RFID microchip. In this way, subject-specific information about the distance travelled, running speed and exercise duration of the movement phases was obtained.

### Experimental procedure

The experiment was conducted as a longitudinal dual tracer [^18^F]UCB-H/[^18^F]FDG study with 24 rats included. All rats underwent a chemical BL by injection of bupivacaine and p-arsanilic acid. Sequential whole brain PET imaging with [^18^F]UCB-H^81^ and [^18^F]FDG combined with computer tomography (CT) imaging was performed at baseline and 1, 3, 5, 7 and 9 weeks post BL (see Supplementary Fig. S2 for details). In the respective weeks, [^18^F]UCB-H imaging was done first, followed by [^18^F]FDG imaging with an interscan interval of > 24 h. All animals underwent behavioural testing by instrumental analysis of locomotion and spatial orientation in the open field at baseline and 1, 2, 3, 5 days, as well as 1, 2, 3, 4, 5, 6, 7, 8, 9 weeks after BL.

### Chemical bilateral labyrinthectomy

Chemical labyrinthectomy was performed as described previously^82,83^: Perioperative analgesia was ensured by pre-emptive subcutaneous administration of meloxicam (1 mg/kg) s.c. 30 min before the procedure. After initiation of the anesthesia with 2-% isoflurane in O2 (1–2 l/min) via a mask, local anesthesia with 0.5% bupivacaine solution (500 µl) was applied s.c. approximately 1 cm dorsomedial of the ear. A double-sided injection of 2.5 ml saline solution into the knee fold was applied to stabilize circulation during surgery. For infection prophylaxis, marbofloxacin was administered s.c. at a dosage of 2 mg/kg. With a paramedian incision, the surgical field was opened, exposing the lamboidal ridge and the external auditory canal. After opening the external auditory canal anterior to the exit point of the facial nerve, the tympanic membrane was perforated caudally to the hammer shaft with a 26-gauge needle. Afterwards 20% bupivacaine solution (150 μl) was injected into the tympanic cavity. The substance was then repeatedly applied and aspirated to avoid bagging into the Eustachian tube. The same procedure was repeated with 10% arsanilic acid (150 μl), which was previously shown to induce irreversible toxic damage to the primary sensory cells of the inner ear^84^. The wound closure was followed by skin suture. This procedure was carried out on both sides, starting on the left side. The analgesic and antibiotic supply was continued postoperatively for a further 3 days by administration of meloxicam (2 mg/kg) s.c. twice daily and administration of marbofloxacin (2 mg/kg) s.c. once daily.

### Criteria for exclusion

Animals were excluded from the study if the following symptoms were observed:

- Loss of body weight equal to or more than 20% of the value before BL
- Ulcer of the cornea, which could occur due to an inadvertent lesion of the facial nerve during BL
- Bleeding from the tympanic cavity, which could prevent the diffusion of bupivacaine or arsanilic acid into the inner ear
- Circulatory failure or peracute apnoea with lethal consequences.

Based on these criteria, two animals had to be excluded.

### Instrumental analysis of locomotion and spatial orientation

In all rats, locomotion and spatial exploration behaviour was recorded sequentially 14-times from baseline to 9 weeks post BL in an open field (70 cm × 70 cm × 36 cm) using an automated video tracking system detecting nose, body center and tail (EthoVision® XT 16, Noldus®, Netherlands). Rats could move freely and were recorded for 10 min per run.

The following parameters were selected and evaluated based on the findings of a previous study^65^: mean velocity (cm/s), total number of zone transitions from “border zone” to “center zone” and total number of rotations per run.

### PET-CT-imaging

The animals were anesthetized with 2% isoflurane in O2 (1–2 l/min) via a mask. For the application of the tracers, the lateral tail vein was catheterized (24-gauge) and a bolus of the tracer injected (in 0.5 ml saline) (40 MBq per tracer). The animals were positioned in the PET-CT scanner (nanoPET/CT®, Mediso Medical Imaging Systems®, Budapest, Hungary) and were kept warm with a heating pad. To avoid any passive movement of the head, its position was fixed using a custom-made head-holder. For individual attenuation correction, an x-ray CT scan (9 min in duration) was performed for each measurement. While the [^18^F]UCB-H PET measurements started right after injection, data processing was based on a 30 min time frame starting at 30 min post injection^85^. [^18^F]FDG scans were acquired from 30 to 60 min post injection and data were analysed for this 30 min time frame. In this setting, animals were allowed to wake up for 15 min after tracer injection before PET-CT scan was initiated.

### Image processing and statistical analysis

The PET reconstruction procedure was an Ordered Subsets Expectation Maximization (OSEM-3D) algorithm with decay correction, scatter correction, attenuation correction, dead time correction, and sensitivity normalization (Mediso Medical Imaging Systems®, Budapest, Hungary). A CT scan was used for attenuation correction. The resulting images had 212 × 212 × 235 voxels of 0.4 × 0.4 × 0.4 mm^3^. Activity distributions were used as a surrogate for cerebral synaptic density in the case of [^18^F]UCB-H^85^ and as a surrogate for cerebral glucose consumption in the case of [^18^F]FDG scans. The volume containing the brains in the images was cropped and rigidly registered into PX Rat atlas space (W. Schiffer)^86^ in PMOD medical image analysis software (PMOD Technologies LLC, RRID: SCR_016547, v4.004). To achieve comparability, normalization to the whole brain mean activity was performed after applying a 0.4 mm isotropic Gaussian filter, using a brain mask in atlas space in a self-written Python script. Subsequently, the images were segmented into brain regions using Px Rat (W. Schiffer) atlas and mean whole brain normalized activity values for every brain region were extracted. In addition, regions of interest (ROI) for the left and right vestibular nucleus were defined. Mean normalized activity values were extracted for the left and right vestibular nucleus ROI and included in the further analysis.

### Statistics

Statistical analysis was performed with IBM SPSS 25 software and Microsoft Excel. Mean whole brain normalized activity values were compared separately for [^18^F]UCB-H and [^18^F]FDG per segmented ROI by one-way repeated measurements ANOVA with post-hoc paired t-tests and Bonferroni correction for multiple testing (baseline, 1, 3, 5, 7, 9 weeks post BL), to delineate regions with significant changes of synaptic density or rCGM following BL. Additionally, voxel-wise analysis based on t-tests was performed in SPM 8 software (Wellcome Department of Cognitive Neurology, Great Britain) between measurements at baseline and 1, 3, 5, 7, 9 weeks post BL for sake of visualization. For PET data, *p*-values < 0.005 were considered significant. Statistical differences of behavioural parameters were determined by one-way repeated measurements ANOVA with post-hoc paired t-tests and Bonferroni correction.

### Animal ethics statement

In this study, we observed humane principles, respected the welfare of animals and excluded situations when animals were in pain.

## Supplementary Information

Supplementary Information.

## Data Availability

The datasets generated for this study are available on request to the corresponding author.
