# Supplementary material for: Longitudinal [18]UCB-H/[18F]FDG imaging depicts complex patterns of structural and functional neuroplasticity following bilateral vestibular loss in the rat
Source: Sci Rep. 2022 Apr 11;12:6049. doi: 10.1038/s41598-022-09936-w (PMC9001652; doi:10.1038/s41598-022-09936-w)
Supplement: Supplementary file 1 — Supplementary Information. [file 41598_2022_9936_MOESM1_ESM.pdf]

Longitudinal [^{18}]UCB-H/[^{18}F]FDG imaging depicts complex patterns of structural and functional neuroplasticity following bilateral vestibular loss in the rat

Melissa Antons[1], Magdalena Lindner[1,2], Maximilian Grosch[1], Rosel Oos[2], Giovanna Palumbo[2], Matthias Brendel[2], Sibylle Ziegler[2], Peter Bartenstein[2], Marianne Dieterich [1,3,4], Andreas Zwergal[1,3]

[1] German Center for Vertigo and Balance Disorders, DSGZ, University Hospital of LMU Munich, 81377 Munich, Germany

[2] Department of Nuclear Medicine, University Hospital of LMU Munich, 81377 Munich, Germany

[3] Department of Neurology, University Hospital of LMU Munich, 81377 Munich, Germany

[4] Munich Cluster of Systems Neurology, SyNergy, Munich, Germany

Corresponding author:

Melissa Antons, German Center for Vertigo and Balance Disorders, DSGZ, University Hospital of LMU Munich, 81377 Munich, Germany, Phone 0049-89-4400-74641,

E-Mail: Melissa.antons@med.uni-muenchen.de

SUPPLEMENTARY FIGURES

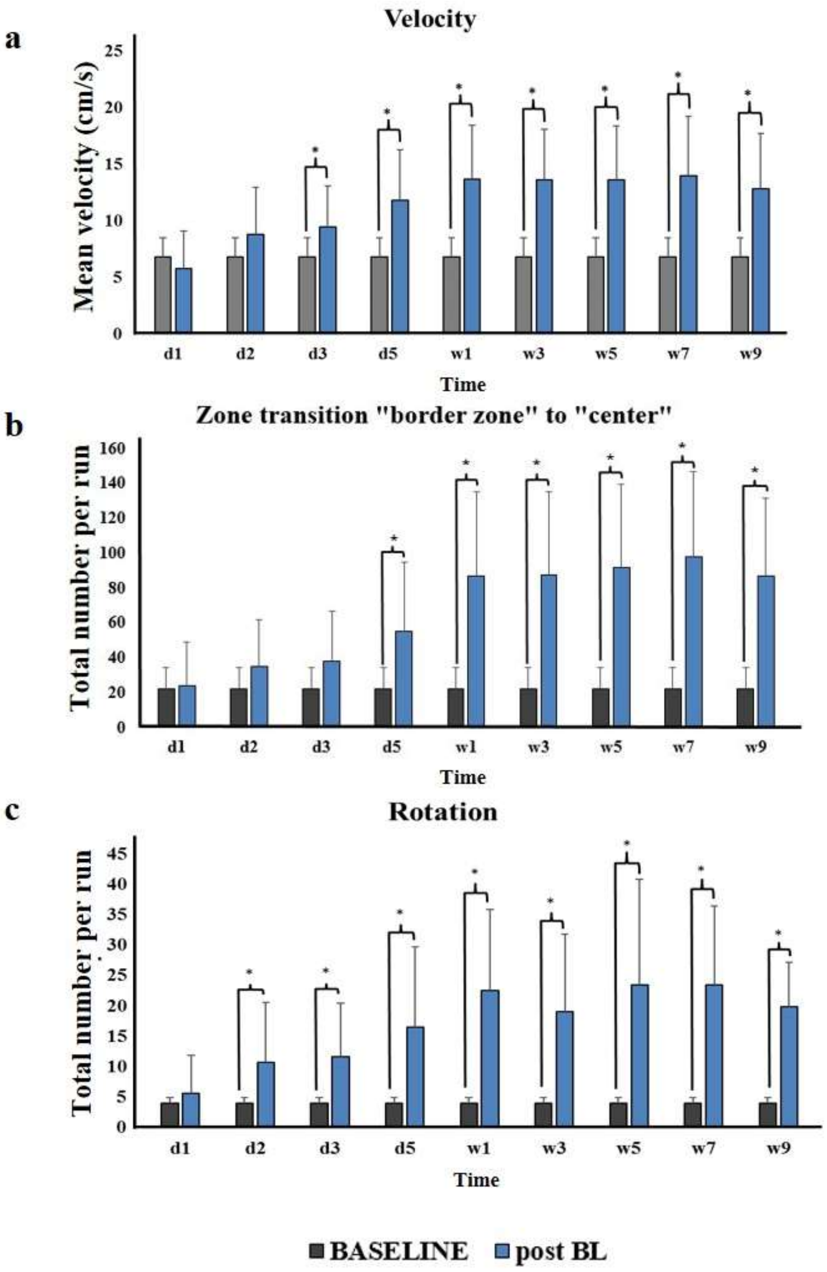

Supplementary Figure S1: **Behavioural parameters for the entire group of rats in the open field.** a) Locomotor velocity from baseline to 9 weeks post BL. b) Total number of zone transition from border to center zone at different time points post BL compared to baseline. c) Total number of rotations per run at different time points post BL. Values are depicted as mean + standard deviation. Significant differences (*) are derived from an ANOVA with post-hoc testing and Bonferroni correction at a level of $p < 0.05$. BL: bilateral labyrinthectomy, d: day, w: week.

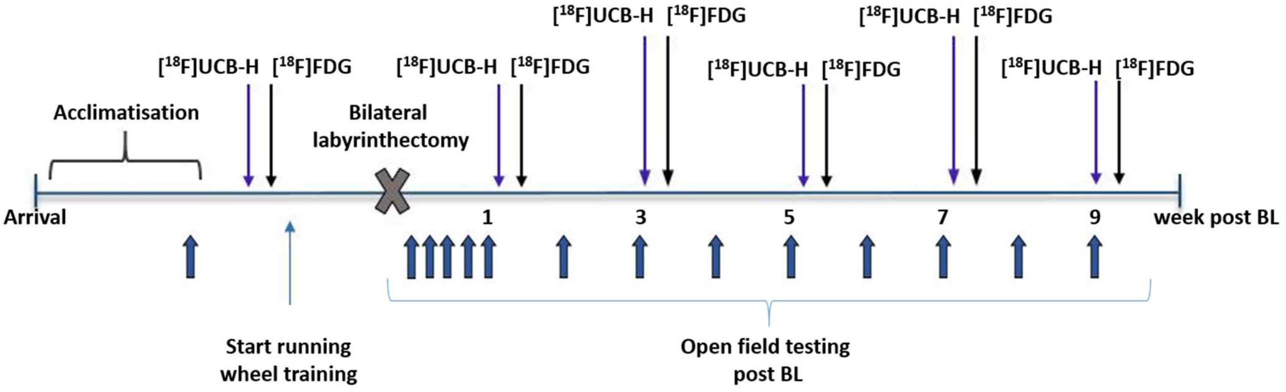

Supplementary Figure S2: **Experimental protocol.** BL: bilateral labyrinthectomy, [^{18}F]FDG: [^{18}F]fluorodeoxyglucose
